# Screening of Lipid-Reducing Activity and Cytotoxicity of the Exometabolome from Cyanobacteria

**DOI:** 10.3390/md22090412

**Published:** 2024-09-10

**Authors:** Rúben Luz, Rita Cordeiro, Vítor Gonçalves, Vitor Vasconcelos, Ralph Urbatzka

**Affiliations:** 1 Faculdade de Ciências e Tecnologia, Universidade dos Açores, 9500-321 Ponta Delgada, Portugal; rita.ip.cordeiro@uac.pt (R.C.); vitor.mc.goncalves@uac.pt (V.G.); 2 CIBIO, Centro de Investigação em Biodiversidade e Recursos Genéticos, InBIO Laboratório Associado, BIOPOLIS Program in Genomics, Biodiversity and Land Planning; UNESCO Chair—Land Within Sea: Biodiversity & Sustainability in Atlantic Islands, Universidade dos Açores, 9500-321 Ponta Delgada, Portugal; 3 Interdisciplinary Centre of Marine and Environmental Research—CIIMAR/CIMAR, University of Porto, Terminal de Cruzeiros do Porto de Leixões, Av. General Norton de Matos s/n, 4450-208 Matosinhos, Portugal; vmvascon@fc.up.pt (V.V.); rurbatzka@ciimar.up.pt (R.U.); 4 Department of Biology, Faculty of Sciences, University of Porto, 4069-007 Porto, Portugal

**Keywords:** zebrafish, anti-steatosis, lipid reduction, exudate, extracellular compounds, flavonoids, chemodiversity

## Abstract

Cyanobacteria are rich producers of secondary metabolites, excreting some of these to the culture media. However, the exometabolome of cyanobacteria has been poorly studied, and few studies have dwelled on its characterization and bioactivity assessment. In this work, exometabolomes of 56 cyanobacterial strains were characterized by HR-ESI-LC-MS/MS. Cytotoxicity was assessed on two carcinoma cell lines, HepG2 and HCT116, while the reduction in lipids was tested in zebrafish larvae and in a steatosis model with fatty acid-overloaded human liver cells. The exometabolome analysis using GNPS revealed many complex clusters of unique compounds in several strains, with no identifications in public databases. Three strains reduced viability in HCT116 cells, namely Tolypotrichaceae BACA0428 (30.45%), Aphanizomenonaceae BACA0025 (40.84%), and Microchaetaceae BACA0110 (46.61%). Lipid reduction in zebrafish larvae was only observed by exposure to *Dulcicalothrix* sp. BACA0344 (60%). The feature-based molecular network shows that this bioactivity was highly correlated with two flavanones, a compound class described in the literature to have lipid reduction activity. The exometabolome characterization of cyanobacteria strains revealed a high chemodiversity, which supports it as a source for novel bioactive compounds, despite most of the time being overlooked.

## 1. Introduction

Cyanobacteria are rich producers of bioactive secondary metabolites [1]. The best-known metabolites are toxins due to their environmental damage associated with cyanobacterial blooms [2]. However, many other metabolites of interest are produced by cyanobacteria, such as dolastatin [3], cryptophycin [4], and even toxins, such as saxitoxins [5], that have long been recognized for their high biotechnological potential. The high chemical diversity and their genomic potential [6,7,8] make cyanobacteria an optimal model for the search for new compounds.

From the wide array of secondary metabolites produced by cyanobacteria, many have been shown to be secreted into the external environment. Examples include polysaccharides [9,10], proteins [11], glycolipids [12], or fatty acids [13]. This secretion mechanism plays a role in granting an ecological advantage to cell survival against foreign agents, protecting against desiccation [14], UV radiation [15], reactive oxygen species [10,16,17], as well as aiding in motility and/or adhesion [18,19,20] or in metal-deficient environments by the production of siderophores [21]. Cyanobacteria is also known to produce allelochemicals with toxic effects [22] or as phytohormones, specifically growth promoters [23]. The transfer of compounds to the extracellular media is mediated through secretory portals or extracellular vesicles [24].

The exometabolome of cyanobacteria is a group of metabolites produced by cells and expelled to the media. It is mainly composed of exopolysaccharides (EPS), which play an important role in biofilm production, providing protection against environmental agents such as UV radiation, desiccation, and predators [25]. Many of the compounds found in the exometabolome are bioactive and may have potential biotechnological applications [25,26], but toxic and teratogenic effects have also been observed in zebrafish (*Danio rerio*) larvae [27,28]. For exopolysaccharides, mainly antioxidant and anti-inflammatory activities were described [10,29,30]. For non-EPS compounds, several studies have reported toxic effects, such as for portoamides [31,32], antifungal activity for tolybyssidins [33], and unidentified compounds with possible anticancer activity [34].

There is a worldwide need to address the increasing incidence of obesity and its comorbidities [35]. Obesity is associated with increased morbidity and mortality [36], and it is a leading factor in the emergence of chronic diseases that cause severe health risks, such as cardiovascular diseases, diabetes [37], and cancer [35,38]. Another related pathology is nonalcoholic fatty liver disease, which causes an abnormal accumulation of fat in liver tissue that can lead to cirrhosis or hepatocellular carcinoma [39]. This fosters an increasing need for treatments to prevent the worsening of obesity-associated pathologies [40]. There has been a significant rise in anti-obesity drugs [41], with numerous studies highlighting the potential of cyanobacteria for obesity treatment [42] through both in vitro [43,44,45] and in vivo approaches [46,47,48]. In human trials, the commonly consumed cyanobacteria Spirulina (*Limnospira* spp.) has been associated with the reduction in triglycerides and total cholesterol [49].

One of the most problematic comorbidities of obesity is cancer [35]. Cyanobacteria have long been associated with cytotoxic activity [50,51,52], with many identified cytotoxic compounds from cyanobacteria (e.g., cryptophycin [4], dolastatin [3], portoamides [53], and leptochelins [54]); many of these are already in commercial use as anticancer drugs, such as dolastatin [55]. However, only a few compounds can also be found in the cyanobacterial exometabolome (for example, portoamides) [32], with few works focusing on these extracts [56] and a small subset of them using purified compounds [53,57,58,59].

This work focuses on a seldom-investigated aspect of cyanobacteria secondary metabolites, the secreted compounds known as exometabolomes. The main aim of this work was to explore the chemodiversity of the cyanobacterial exometabolome of 56 cyanobacterial strains from the Bank of Algae and Cyanobacteria of the Azores (BACA) culture collection and to identify possible bioactive strains. Exudate extracts were tested for cytotoxic effects against two cancer cell lines, HepG2 and HCT116, to reveal potential anticancer activities. In addition, exudates were tested for their reduction in lipids in an antisteatosis model using the HepG2 cell line in vitro and in the fat metabolism assay in zebrafish larvae. Metabolomic profiling of the cyanobacterial extracts was performed on the Global Natural Product Social Molecular Networking (GNPS) platform to characterize the chemodiversity and to identify unique mass features in the bioactive strains, which could be related to the observed bioactivity.

## 2. Results

### 2.1. Bioactivity Results

All 56 cyanobacteria produced extracellular compounds; however, in very different amounts, ranging from around 0.05 g to 1.5 g of dry weight of the exudates from each 400 mL of media (Table S1). The extracts were applied in in vitro assays for cytotoxicity in HepG2 and HCT116 cell lines, lipid reduction using the HepG2 cell line as an antisteatosis model, and zebrafish larvae for the in vivo model.

**Figure 1:**
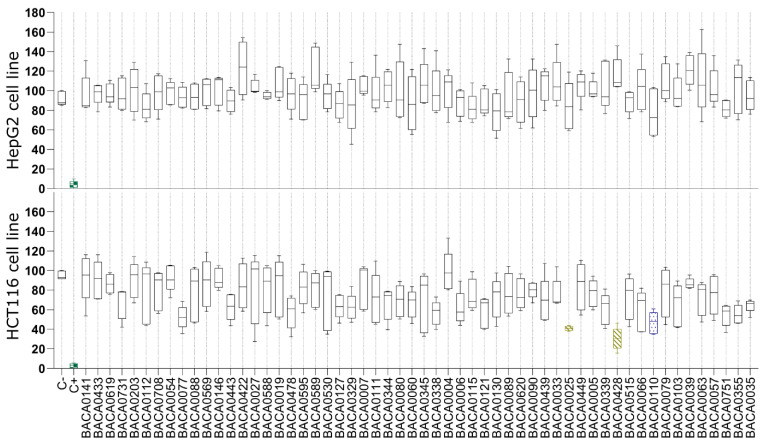
Cell viability after 48 h of exposure to cyanobacterial exudate extracts at 25 µg/mL in the HepG2 and HCT116 cell lines. Strains are taxonomically sorted. Statistical differences vs. the solvent control are indicated in color: green: *p* < 0.001; yellow: *p* < 0.01; blue: *p* < 0.05, Kruskal–Wallis test followed by Dunn’s posthoc test.

In the cytotoxicity assays (Figure 1), none of the extracts significantly reduced the viability of the HepG2 cells. On the contrary, exposure to extracts of three strains resulted in low cellular viability on the HCT116 cell line, namely: Tolypotrichaceae BACA0428 (30.45%), Aphanizomenonaceae BACA0025 (40.84%), and Microchaetaceae BACA0110 (46.61%).

**Figure 2:**
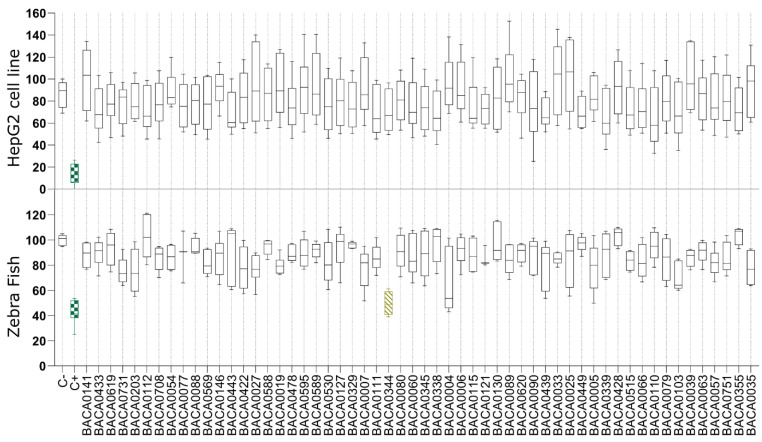
Lipid reduction in the steatosis model with fatty acid overloaded HepG2 cells and in zebrafish larvae exposed to cyanobacterial exudate extracts at 25 µg/mL. Strains are taxonomically sorted. Statistical differences vs. the solvent control are indicated in color, green: *p* < 0.001; yellow: *p* < 0.01, according to the Kruskal–Wallis test followed by Dunn’s posthoc test.

**Figure 3:**
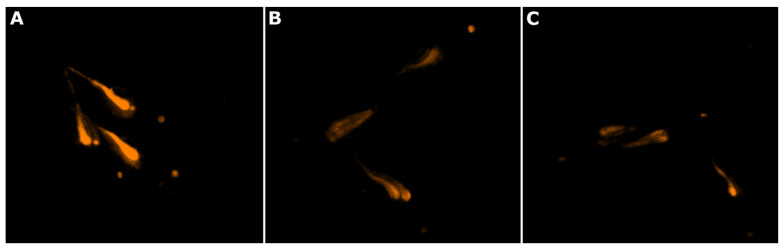
Reduction in neutral lipids in zebrafish larvae, stained with Nile red (orange), by the exudate extract. (**A**) zebrafish larvae from the negative control group (water); (**B**) zebrafish larvae exposed to resveratrol at 50 µM (positive control); (**C**) zebrafish larvae exposed to *Dulcicalothrix* sp. BACA0344 exudate extract.

In the antisteatosis model, no statistically significant differences were observed for any strain vs. the negative control group (Figure 2). In contrast, exposure to the exudate extract from *Dulcicalothrix* sp. BACA0344 resulted in significantly less fluorescence in zebrafish larvae when compared to the negative control (Figure 3), supporting a strong reduction in neutral lipids.

### 2.2. Feature-Based Molecular Network Analysis

**Figure 4:**
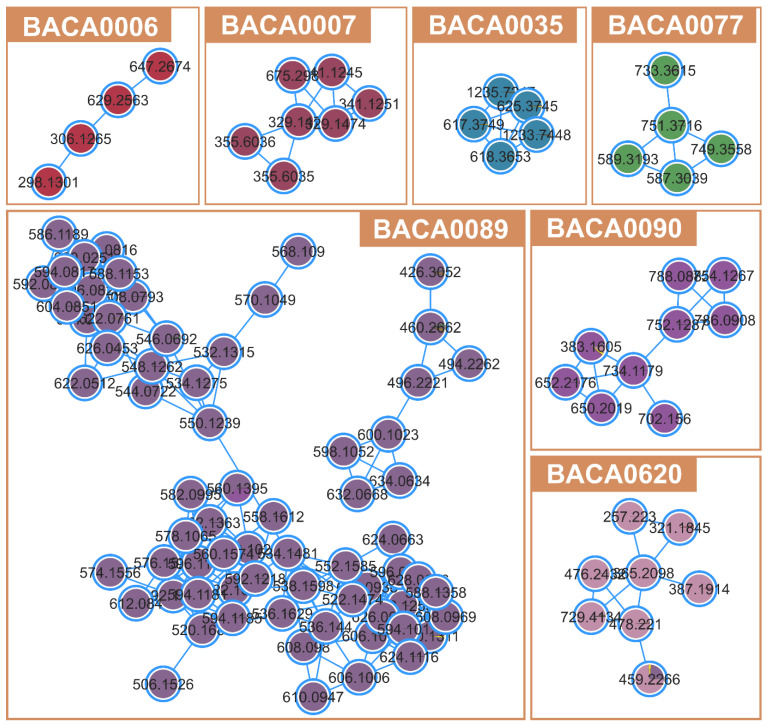
Unidentified clusters from FBMN belonging to non-bioactive strains.

The metabolite network (1843 compounds) of the 56 studied strains is presented in File S1. The FBMN revealed several clusters to be produced by just one strain (Figure 4). No putative identifications were obtained for such clusters, which could represent novel compounds. The biggest clade was produced by *Neowestiellopsis persica* BACA0089, and although no bioactivity was identified, it contributed the most to the molecular network (Figure 4). Other strains creating relevant clades of secondary metabolites (≥4 compounds) are *Phayaothrix* sp. BACA0006, *Kamptonema* sp. BACA0007, *Aliinostoc* sp. BACA0035, *Pegethrix* *atlantica* BACA0077, *Symphyonema* sp. BACA0090, and Hapalosiphonaceae BACA0620.

**Figure 5:**
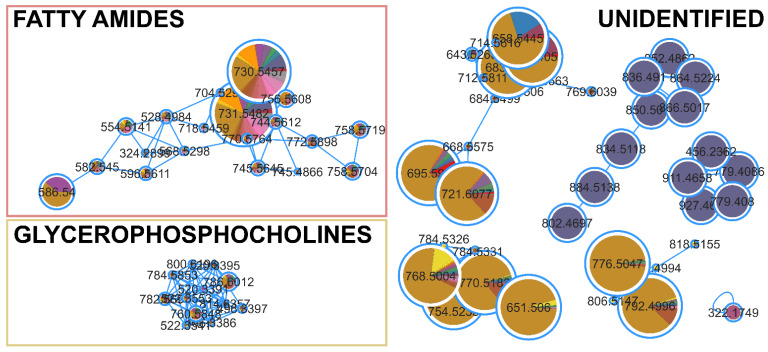
Clusters from the FBMN for cytotoxic activity against HCT116 cell lines. Significantly correlated nodes are highlighted with size and color (Aphanizomenonaceae BACA0025 in purple; Microchaetaceae BACA0110 in pink; Tolypotrichaceae BACA0428 in dark yellow).

The FBMN analysis for cytotoxicity against the HCT116 cell line identified 51 compounds (Table S2) with significant correlation (*p* < 0.05, correlation > 0.5). The identified clusters consisted of fatty amides, glycerophosphocholines, and many unidentified clusters of compounds (Figure 5), mainly produced by Tolypotrichaceae BACA0428 or Aphanizomenonaceae BACA0025. Microchaetaceae BACA0110 did not have correlations in any cluster, but some single nodes with low correlation and no putative identifications.

**Figure 6:**
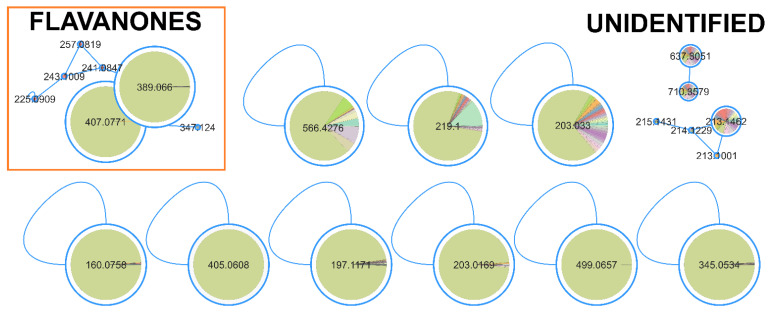
Clusters from the FBMN for lipid reduction activity in zebrafish larvae. Significantly correlated nodes are highlighted with size and color (*Dulcicalothrix* sp. BACA0344 in green).

For lipid reduction in the zebrafish larvae, only the strain *Dulcicalothrix* sp. BACA0344 demonstrated activity. The FBMN analysis identified 18 compounds (Figure 6; Table S2) correlated with lipid-reducing activity, and 14 of them with a very high correlation (>0.9). Although many compounds are represented by unclassified single nodes in GNPS or MS2Query, two compounds related to the bioactivity were present in a cluster and identified as flavanones.

## 3. Discussion

The BACA culture collection is a highly taxonomically diverse culture collection [60], containing many newly described genera and species [61,62,63,64]. Focusing on the exometabolome, 56 strains of cyanobacteria from this collection were studied, offering a vast array of genetic resources for this research. Cyanobacteria culture medium is normally rich in EPS [65], characterized by a high variability of excreted compounds [65,66] and as a source of bioactive metabolites [31,32,67,68]. However, extracts from supernatants are less complex than biomass-derived extracts, which provides advantages for isolating possible new compounds. The studied extracts resulted in a total of 1843 mass features with MS2 data. From these, using the MS2 data, 61 (3%) were identified using the GNPS, and class prediction was made for 203 (11%) using MS2Query. Using only MS1 (*m/z*), it was possible to identify 85 (5%) masses using the CyanoMetDB and 519 (28%) using the NPA both within a deviation of <5 ppm. Although identifications using NPA or CyanoMetDB must be taken with caution, the low percentage of identified metabolites highlights the high number of compounds still to be characterized.

A high variability in exudate production (Table S1) was observed, ranging from around 0.05 g to 1.5 g. Strongly bioactive strains had a low production of exudate (0.05–0.1 g), which could be related to a lower production of EPS. Albeit EPS are often mentioned in the literature with several bioactivities [69], it can be produced in very high amounts and conceal non-EPS bioactive compounds when used in low extract concentrations, as performed in this work. This can be observed in Figure 4, where bigger compound clusters were only detected from strains with low exudate production. Several BACA strains presented complex unclassified clusters of compounds (e.g., *Neowestiellopsis persica* BACA0089 and *Symphyonema* sp. BACA0090), which represent a big diversity of the cyanobacterial exometabolome still to characterize. Also, the observed clusters in Figure 4 are related to the presented strain, meaning an exclusive compound production, reinforcing the high chemodiversity of cyanobacteria at the strain level. Compound production appears to be more influenced by taxonomy than by the habitat of origin, with Nostocales strains showing the greatest compound diversity, regardless of the biotopes from where they were isolated.

The BACA0025 strain is a novel yet undescribed genus known for producing cylindrospermopsin [70]. However, this compound was not detected in the analyzed exometabolome. In the FBMN (Figure 5), BACA0025 exhibited several clusters unique to the strain. One of these clusters had a putative identification from NPA on the mass feature 866.5017 *m/z* as Anabaenopeptin T, a carboxypeptidase A inhibitory compound, with an error margin of 1.20 ppm [71,72]. Despite GNPS and MS2Query not identifying any similar compounds in the clade, this might be an Anabaenopeptin-related clade. However, none of the other masses in the cluster were matched with any known compound using either NPA or CyanoMetDB. The other putative identification, also in the cluster presented in Figure 5, is the mass of 927.4609 *m/z*, putatively identified as Nodularin-R-phenylglyoxal (IIa/b) with an error of 0.74 ppm. Nodularin-R-phenylglyoxal (IIa) is a Nodularin-R synthetic derivative that induced apoptosis in microinjected Swiss 3T3 fibroblasts [73], although the capacity of the strain to produce this analog is uncertain. However, no microcystins/nodularin genes were amplified, nor was the toxin identified, in this case MC-LR [70]. The presence of a complete cluster produced exclusively by BACA0025, which was correlated with the bioactivity, supports the use of this strain in the search for new bioactive extracellular compounds. BACA0428 showed the strongest cytotoxicity against HCT116. The metabolite profiling by FBMN showed many metabolites, mostly produced by this strain evidencing specific clusters with several compounds, though none had putative identifications. Nonetheless, this suggests that cyanobacterial exudate might be a rich source of bioactive secondary metabolites. Considering the diversity of cyanobacteria in the BACA culture collection, many new compounds could be discovered.

Lipid reduction in zebrafish larvae is used as a model for metabolomic diseases, such as obesity, due to the functional conservation in lipid metabolism and adipose biology, among others [74]. There are many known natural compounds with lipid reduction potential [75], such as resveratrol, a well-characterized polyphenol [46,76], used as the positive control in this work. A cluster of flavanones was identified from *Dulcicalothrix* sp. BACA0344 (Figure 6), having two masses of 389.066 *m/z* and 407.0771 *m/z* with high correlation, though none matches known compounds in the searched databases. Flavanones are a class of flavonoids derived from the chalcone structure, containing three rings [77]. Flavonoids have been related to several bioactivities, mainly antioxidant and anti-UV protector [78,79]. In the zebrafish larvae model, several commercially used flavonoids (e.g., flavone, kaempferol, quercetin, among others) presented a strong reactive oxygen species scavenging rate at a concentration of 20 ppm [79]. In lipid-reducing assays using zebrafish larvae exposed to extracts of wine lees with a high concentration of flavonoids (mainly rutin and quercetin), at a concentration of 100 µg/mL, it proved to have a 40% reduction with significant effects on expression of lipid metabolism key genes, such as FASN and CPT1B [80]. Reduction in lipids was also observed in zebrafish larvae fed with a high-fat diet from 5 dpf to 20 dpf while exposed to 20 μM kaempferol [81]. Similarly, zebrafish exposed to 6.25 uM Baicalein, grown under the same conditions, exhibited a 30% reduction in lipid accumulation [82]. The described bioactivity of flavonoids in the literature supports the search for new bioactive flavanones excreted from the culture medium. Cyanobacteria, and in this case *Dulcicalothrix* sp. BACA0344, have demonstrated to be a promising source for novel lipid-reducing compounds. However, this bioactivity appears to be species or strain-dependent, as the other two studied *Dulcicalothrix* sp. strains (BACA0060 and BACA0080) did not yield positive results. Therefore, further research should focus on BACA0344.

## 4. Materials and Methods

### 4.1. Biomass and Extract Production

Fifty-six uni-cyanobacterial strains from the BACA culture collection were selected according to habitat and phylogenetic diversity (Figure S1), including freshwater (aquatic and terrestrial), brackish, and thermal cyanobacteria from 11 taxonomic orders (Table S1). For the phylogenetic analysis, the 16S rRNA sequences were selected according to the current literature and retrieved from GenBank together with published BACA sequences. These were aligned using MAFFT v 7.520 [83] and phylogenetic analysis using maximum likelihood, with 1000 ultrafast bootstrap [84], in the IQ-Tree online version v1.6.12 [85] with the model, TVMe + I + G4 chosen according to the Bayesian Information Criterion, using ModelFinder [86].

Cyanobacteria cultures were grown in 1 L flasks with aeration for four weeks with a 14:10 h light:dark cycles, at an intensity of approximately 110 µmol/s/m^2^ of light and temperature of 25 °C. Biomass was centrifuged at 15,000× *g* for 10 min. A total of 400 mL of supernatant was recovered and filtered with 1.2 µm glass fiber filters, except for BACA0019 and BACA0588, when the supernatant was filtered with 0.7 µm glass fiber filters due to the lower cell size. The supernatant was frozen and freeze-dried at −45 °C and 0.1 mBar. The produced freeze-dried extracts were resuspended in ultra-pure water for further use.

### 4.2. Bioactivity Assays

For the cytotoxicity assessment, HepG2 and HCT116 cells were used from the American Type Culture Collection (ATCC) (Manassas, VA, USA). Cells were cultured in Dulbecco Modified Eagle Medium (DMEM) (Gibco, Thermo Fisher Scientific, Waltham, MA, USA) supplemented with 10% (*v/v*) fetal bovine serum (FBS) (Biochrom, Berlin, Germany), 1% penicillin/streptomycin (100 IU/mL and 10 mg/mL, respectively) (Biochrom), and 0.1% amphotericin B (GE Healthcare, Little Chalfont, UK).

The phenotypic antisteatosis assay was performed as described by Costa et al. [87]. Briefly, HepG2 cells were seeded at 1 × 10^5^ cells/well, and after 24 h, the medium was changed to DMEM without FBS, supplemented with 62 µM sodium oleate (Sigma-Aldrich, St. Louis, MO, USA), and exposed to extracts at 25 µg/mL. Positive control was exposed to DMEM without FBS, supplemented with 62 µM sodium oleate, and negative control to DMEM without FBS and sodium oleate supplementation. After 6 h, 75 ηg/mL Nile red (Sigma-Aldrich) and 10 µg/mL Hoechst 33342 (HO-33342) (Sigma-Aldrich) were added and incubated for 15 min at 37 °C in the dark. Fluorescence images were taken with a BioTek Cytation 5 Cell Imaging Multimode Reader (Santa Clara, CA, USA) at 531/593 nm (RFP) excitation/emission for Nile Red and 377/447 nm (DAPI) excitation/emission for HO-33342. DAPI and RFP images were overlapped, and threshold analysis was carried out to determine the percentage of lipid-positive cells compared to the negative control. For the cytotoxicity analysis, the MTT assay was used as previously described [88]. Both HCT116 and HepG2 cells were seeded at 3.3 × 10^4^ cells per well with medium renewal at 24 h, and cells exposed to the extracts at 25 µg/mL for 48 h.

The lipid reduction in zebrafish (*Danio rerio*; WT AB strain) larvae was carried out as described by Costa et al. [87] using the Nile red fat metabolism assay. Larvae were raised in E3 medium at 28 °C with 200 µM 1-phenyl-2-thiourea (PTU). At 3 days post-fertilization (DPF), the larvae were exposed to extracts at a concentration of 25 µg/mL. Ultra-pure water and resveratrol (REV, at 50 µM) were used as solvents and positive controls, respectively. For imaging, the larvae were stained overnight with Nile red at 10 ng/mL. Imaging was performed with the BioTek Cytation 5 Cell Imaging Multimode Reader (Santa Clara, CA, USA), and the fluorescence intensity was quantified at 531/593 nm (RFP) excitation/emission in each individual zebrafish larvae using ImageJ v1.54d [89].

### 4.3. Statistical Analysis

All screenings were performed in two independent assays in triplicates per sample (*n* = 6). The Gaussian distribution of data was tested by a Kolmogorov–Smirnov normality test (*p* < 0.05). One-way ANOVA was used followed by Kruskal–Wallis with Dunn’s post hoc test (non-parametric distribution). Statistically significant differences were considered with *p* < 0.05. All statistical analyses were carried out in GraphPad Prism v9.5.0.

### 4.4. Exometabolome and Feature-Based Molecular Networking

The dried extracts were desalted using Pierce™ C18 Tips, following the recommended protocol for LC-MS/MS analysis, with a final elution made with 0.1% formic acid in 95% methanol LC-MS/MS grade. Extracts were then analyzed using liquid chromatography-high resolution electrospray ionization tandem mass spectrometry (HR-ESI-LC-MS/MS) on a Q Exactive Focus Orbitrap LC-MS/MS system (Thermo Fisher) in the positive mode at the Materials Centre of the University of Porto, as previously described in detail [88].

The feature-based molecular network (FBMN) analysis followed previously described conditions [90]. Briefly, raw data were converted to .mzML format files using MSConvert [91], and data were processed with MZmine v2.53 [92]. The FBMN workflow ran on the GNPS platform [87] with default parameters after MZmine processing. Data were visualized using Cytoscape v3.10.2 [93].

Molecular class enrichment and cluster class classification were conducted using MS2Query [64] run locally and molDiscovery [94] on the GNPS platform. For the MS2Query, the MS2 data (.mgf file output from FBMN) was analyzed using MS2Query v1.3.0 [95], in positive mode, using the available pre-trained embeddings and models [96], with analogs considered only with ≥0.7 MS2QueryScore as thresholds with compound class prediction by NPClassifier [97]. The FBMN output was also used for molDiscovery v1.0.0, which ran on the GNPS platform with the default database. The workflow ran with a custom value of precursor ion mass tolerance set to 5 ppm and fragment ion mass tolerance set to 0.02 Da. Compound class prediction was made using NPClassifier [67].

Statistical analysis followed the script made available by Nothias et al. [90] (v1.1 for R) for calculation of the *p*-value (after Bonferroni correction) and Pearson correlation (PC) between feature intensity and bioactivity level. Mass peaks with *p* < 0.05 and correlation > 0.50 were matched to the Natural Product Atlas (NPA) database v2024_03 [98] and CyanoMetDB v0.2.0 [99] within 5 ppm for a putative identification, using a custom-made R script with compound class prediction based on NPClassifier [97].

## 5. Conclusions

This work comprised a wide bioactivity prospection on cultured cyanobacteria from the Azores Islands and identified several bioactive strains from the BACA culture collection with the production of extracellular bioactive compounds. The large unidentified chemodiversity on the cyanobacterial exometabolome supports the need for further studies. Strong cytotoxic activity from Aphanizomenonaceae BACA0025, Microchaetaceae BACA0110 and Tolypotrichaceae BACA0428 was observed, and from the analyzed FBMN, several unique clusters of compounds were identified, mainly fatty amides. However, the majority are unidentified clusters, reinforcing the presence of possible novel bioactive compounds. In the lipid reduction assay with zebrafish larvae, the exudates of *Dulcicalothrix* sp. BACA0344 had strong activity, and several mass peaks were correlated and putatively identified as flavanones. Cyanobacteria have not been extensively studied for their flavonoid production and diversity, suggesting the potential discovery of new bioactive flavanones. This reinforces the importance of exploring cyanobacteria for novel lipid-reduction compounds. The presence of many bioactive compounds on the cyanobacteria exometabolome also supports future biorefinery approaches, allowing full use of cyanobacterial cultures, from biomass to the waste media of cyanobacterial production.

## Data Availability

The raw data supporting the conclusions of this article will be made available by the authors on request.

## Supplementary Materials

The following supporting information can be downloaded at: https://www.mdpi.com/article/10.3390/md22090412/s1, File S1: Cytoscape file with the feature-based molecular network with annotated clusters; Figure S1: Phylogenetic analysis of the 16S rRNA gene of the studied strains and sequences retrieved from the current literature. Used BACA strains are presented in bold; Table S1: List of used cyanobacteria strains from the Bank of Algae and Cyanobacteria of the Azores culture collection with taxonomic data, habitat description and extracts yield; Table S2: List of statistically significant compounds associated with lipid reduction in zebrafish larvae and cytotoxicity. Match in GNPS, MS2Query, molDiscovery, CyanoMetDB and Natural Product Atlas are presented for each compound.
