# Supplementary figures and images for: Screening of Lipid-Reducing Activity and Cytotoxicity of the Exometabolome from Cyanobacteria
Source: Mar Drugs. 2024 Sep 10;22(9):412. doi: 10.3390/md22090412 (PMC11433081; doi:10.3390/md22090412)

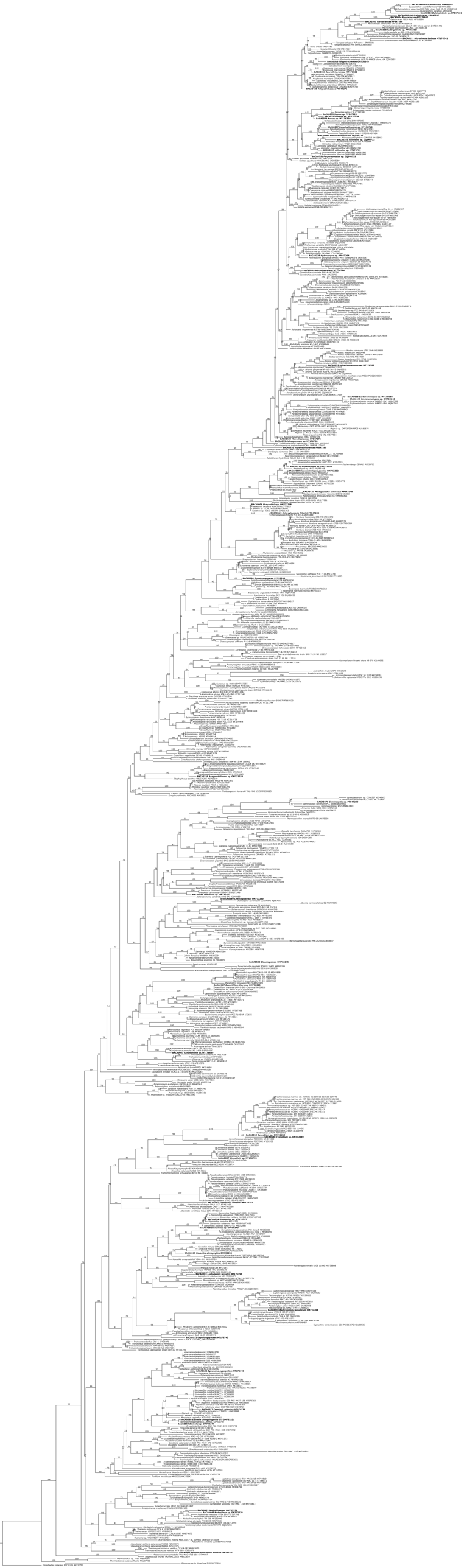

Supplement: Supplementary file 1 [file marinedrugs-22-00412-s001.zip › Fig_S1.pdf]
